# Is this love? Sex differences in dog-owner attachment behavior suggest similarities with adult human bonds

**DOI:** 10.1007/s10071-021-01545-w

**Published:** 2021-08-05

**Authors:** Biagio D’Aniello, Anna Scandurra, Claudia Pinelli, Lieta Marinelli, Paolo Mongillo

**Affiliations:** 1 Department of Biology, University of Naples Federico II, via Cinthia, 80126 Naples, Italy; 2 Department of Environmental, Biological and Pharmaceutical Sciences & Technologies, University of Campania “Luigi Vanvitelli”, Caserta, Italy; 3 Laboratory of Applied Ethology, Department of Comparative Biomedicine and Food Science, University of Padua, Viale dell’Università 16, 35020 Legnaro, PD Italy

**Keywords:** Animal cognition, Attachment, Dog, Human, Sex differences, Strange situation test

## Abstract

Sex differences in the behavioral responses of Labrador Retriever dogs in the Strange Situation Test were explored. Behaviors expressed by dogs during seven 3-min episodes were analyzed through a Principal Component Analysis (PCA). The scores of factors obtained were analyzed with a Generalized Linear Mixed Model to reveal the effects of the dog’s sex and age and the owner’s sex. In Episode 1 (dog and owner) and 5 (dog alone), the PCA identified three and two factors, respectively, which overall explained 68.7% and 59.8% of the variance, with no effect of sex. In Episodes 2 (dog, owner, and stranger), 3 and 6 (dog and stranger), and 4 and 7 (dog and owner), the PCA identified four factors, which overall explained 51.0% of the variance. Effects of sex were found on: Factor 1 (distress), with lower scores obtained by females in Episode 2 and higher in Episode 3; Factor 2 (sociability), which was overall higher in females; Factor 3 (separation-distress), with females, but not males, obtaining higher scores when left with the stranger than when with the owner. Therefore, females were overall more social but seemed more affected than males by the owner’s absence. Parallels can be traced between our results and sex differences found in adult human romantic attachment, suggesting that the dog-owner bond has characteristics that are not found in the infant-mother relationship.

**Supplementary Information:**

The online version contains supplementary material available at 10.1007/s10071-021-01545-w.

## Introduction

Dogs (*Canis lupus familiaris*) show high socio-cognitive skills for interacting with humans, allowing them a better adaptation to the anthropogenic niche (Miklósi and Topál 2013; Udell and Wynne 2008). This ability is acquired both through genetic (Hare et al. 2002; Hare and Tomasello 2005) and ontogenetic (Barrera et al. 2015; Udell et al. 2010, 2011) factors, although their relative weight remains yet unsettled (Passalacqua et al. 2011).

Among the most relevant socio-cognitive skills are the high degree of attention toward humans (Alterisio et al. 2019; Mongillo et al. 2015; Virányi et al. 2004), the ability to learn hundreds of words (Kaminski 2004; Pilley and Reid 2011), and the recognition of human body language as the main source of information (D’Aniello et al. 2016, 2017; Scandurra et al. 2017, 2018a). Besides being skilled in interpreting some forms of human communication, dogs are also able to send effective signals to humans, for example seeking help when encountering an unsolvable problem (D’Aniello and Scandurra 2016; Miklósi et al. 2003; Scandurra et al. 2015). In these communicative exchanges, the ability of dogs to detect and reflect emotional reactions in the sender plays an important role. Indeed, dogs seem to be well apt in detecting human emotions using different sensory modalities, such as visual and acoustic stimuli (Siniscalchi 2018a, b; Turcsán et al. 2015), as well as chemical cues (D’Aniello et al. 2018; Semin et al. 2019; Siniscalchi et al. 2016). The reading of human emotions can drive dogs' behavioral responses in the social referencing process allowing them to approach or avoid a fearful stimulus (Merola et al. 2012a,^2013^, b). It is not clear whether dogs understand the fear emotion conveyed by vocal and visual signals of humans (Yong and Ruffman 2015) but it is established that they perceive the fearful message transferred by chemosignals (D’Aniello et al. 2018; Siniscalchi et al. 2016).

One of the most interesting and peculiar aspects of the dog–human relationship is dogs’ predisposition to form lasting affectional bonds with their human caretakers, which have been equated to the attachment relationship that human infants form towards their mothers (Palestrini et al. 2005; Palmer and Custance 2008; Prato-Previde et al. 2003; Rehn et al. 2013; Topál et al. 1998). This refers to a social bond between parents and offspring, where the latter benefits from the support of their caretakers (Ainsworth 1989). The attachment bond is expressed behaviorally through a preference for one or more specific individuals who are the attachment figures. Proximity- and contact-seeking behaviors to the attachment figure, signs of distress when involuntary separations occur, and the expression of more confident behaviors when the caregiver is present, are key features of attachment (Ainsworth 1989; Ainsworth and Bell 1970; Bowlby 1958, 1969; Bretherton 1992; Rajecki et al. 1978). One of the best-known methods to empirically explore attachment behaviors is the “Strange Situation Test” (SST), introduced to study the infant-mother attachment bond in humans (Ainsworth and Bell 1970; Ainsworth et al. 1978). Indeed, the behavior expressed by dogs in adapted versions of the SST fulfills attachment criteria, including proximity seeking to the owner, distress and protest behavior upon short-term separation from the owner (Prato-Previde et al. 2003; Topál et al. 1998), quickly returning toward the owner in the presence of perceived threats (safe-haven effect: Gácsi et al. 2013) and exploring within a wider range when the owner is present (secure-base effect: Horn et al. 2013; Mariti et al. 2013a; Palmer and Custance 2008).

Whether the attachment bond toward a human caregiver already exists in puppies is unclear. It was observed in 4-month-old puppies (Topál et al. 2005), which showed specific patterns of attachment (proximity seeking upon separation and physical contact upon reunion) for the owner. On the other hand, more recent research showed that 2-month-old puppies did not establish yet an attachment bond towards their human caregivers (Mariti et al. 2020). The phenomenon is quite flexible concerning the dog’s age and to the number of figures the dog can attach to; studies in guide dogs, which change several reference figures until they are finally assigned to the visually impaired owner, showed that previous separation events do not preclude the formation of an attachment bond with a new owner (Fallani et al. 2006, 2007; Valsecchi et al. 2010). Furthermore, dogs living in a human family tend to form a stronger attachment bond to a specific member and the preferred person was who cared more about the dog (Carlone et al. 2019). The ability to establish an attachment bond seems to be unaffected by ontogenesis in adult dogs since no clear differences with pet dogs were found in dogs for search and rescue (Mariti et al. 2013b), guide for visually impaired people (Fallani et al. 2006), and water rescue (Scandurra et al. 2016) training experiences, although in the latter study water rescue training affected some of the behavior recorded in the SST (i.e., individual play). Finally, attachment bonds can develop rapidly also in socially deprived dogs (Gácsi et al. 2001).

One question of relevance is whether differences between dog sexes exist in the expression of attachment bond towards humans. In many species, the two sexes are biologically adapted for different scopes, entailing some behavioral specialization, which often regards social and caretaking relationships; indeed, one of the most common and evident differences between sexes is the higher aptness of females to the care of the offspring, while males are more prone to the defense of the territory while trying to inseminate as many females as possible (Fitzpatrick et al. 1995; Rosvall 2011; Rubenstein and Lovette 2009). It is accepted that differences in sex-specific behavioral traits are driven by sexual selection (Schuett et al. 2010). During domestication, wolves (*Canis lupus lupus*) progressively shifted from natural and sexual selection to artificial selection, lowering selective pressure for essential survival traits on dogs (Lorenzen 2003). Nonetheless, a recent review suggests that sex-based differences in some behavioral and cognitive traits of dogs remained quite unchanged from their ancestors (Cassidy et al. 2017), despite the reduced impact of natural and sexual selection (Scandurra et al. 2018b). For example, previous studies underlined a male advantage in flexibly using spatial information (Fugazza et al. 2016; Mongillo et al. 2017a; Scandurra et al. 2018c), which is an important requisite for male dogs since they range over significantly larger areas than females when free-roaming (Sparkes et al. 2014). Concerning the social sphere, male dogs show generally a higher degree of aggressiveness (Eken Asp et al. 2015; Pérez-Guisado et al. 2006), with the greatest likelihood of occurrence in males especially in contexts aimed to raise reproductive success (Borchelt 1983). This pattern is consistent with the theory of behavioral ecology, predicting that higher levels of aggressiveness have a greater positive outcome for male’s fitness (Andersson 1994). On the other hand, females appear to be more sociable (Lore and Eisenberg 1986; Wilsson and Sundgren 1997), soliciting cooperative behaviors more than males (Persson et al. 2015), which again match with the prediction of behavioral ecology theory (Muller and Mitani 2005; Wrangham and Smuts 1980). Finally, differences between sexes were found in relevant aspects of the dog-owner relationship. In such a context, females displayed more referential gaze than males toward the owner (Duranton et al. 2016; Mongillo et al. 2016), while males had a higher ability to recognize the face of the latter (Eatherington et al. 2020; Mongillo et al. 2017b).

Given these differences in the social sphere, it would not be surprising if males and females showed differences in the way they express the attachment bond to humans. To date, most of the studies that assessed sex effects on dogs’ behavior in the SST, seem to converge on the lack of significant differences between males and females (e.g.,Fallani et al. 2006; Gácsi et al. 2001; Topál et al. 1998). However, all of the previous studies included sex as a potentially confounding factor to be controlled for, rather than planning the experiment to assess this variable primarily, and sampling, procedural and analytical differences may not have been tuned to detect those differences in a population of pet dogs. The present study aimed specifically at exploring differences in behavioral responses of males and females’ dogs in the SST. Considering all the sex differences in dog’s social behavior and cognition, we do expect to find sex effects in the behavior expressed in the SST, although the current knowledge does not allow us to make any prediction on whether these differences could be indicative of a different attachment bond between males and females.

## Material and methods

### Subjects

The subjects were 51 Labrador Retrievers (29 males, age 2.9 ± 2.4 years and 22 females, age 3.5 ± 2.8 years) recruited through the Internet, personal contacts, and advertisements in veterinary clinics. Three males out of 29 were castrated; 10 females out of 22 were ovariectomized. The inclusion criteria for the dogs were a good state of health, without any treatment of physical or behavioral disorders, and the lack of separation-related problems. Female intact dogs were not in the estrous phase. The age of the dogs ranged from 7 months to 13 years old. All dogs lived in apartments, with human families composed of at least two people. The owner sex was quite balanced with dog sex: female dogs had 10 female and 12 male owners; male dogs had 11 female and 18 male owners. About 20% of dogs had some training experience (one search and rescue dog, three water rescue dogs, and six dogs with basic training).

### Experimental procedure

Dogs were tested in an unfamiliar room (about 12 m^2^) using the protocol of the SST, adapted to test the attachment bond in dogs (Prato-Previde et al. 2003). The tests were conducted at the Laboratory of Canine Ethology of the University of Naples Federico II (Naples, Italy). The room contained two chairs (randomly assigned to the owner or the stranger) and a table next to one of the walls. Dog toys (two tennis balls and two plastic bottles) and a water bowl were also present. The scene was recorded by two Sony Handycam video cameras (HDR-CX115 and HDR-PJ260VE) placed in opposite corners of the room.

Dog–human pairs entered a waiting room where the experimenters described the procedure and verbally provided the instructions to the owner to act in line with the procedure, without disclosing the aim of the study. Then, participants were taken to the experimental room. The SST consisted of seven consecutive 3-min episodes for a total duration of 21 min, during which the presence/absence of the person (i.e., owner or stranger) was alternated, as described in Table 1. A male experimenter played the role of the stranger. Both the stranger and the owner were passive toward dogs’ solicitations apart from the social play that was actively proposed to dogs.

**Table 1:** Description of the strange situation test procedure

Episodes	Episodes labels	Description
Episode 1: Dog and Owner	1-DO	The owner sat quietly reading a magazine and the dog was free into the room
Episode 2: Dog, Owner, and Stranger	2-DOS	The stranger entered the room, sat quietly for 1 minute, talked with the owner for the second minute, approached the dog, and attempted to stimulate play during the third minute. At the end of this episode, the owner left the room unobtrusively
Episode 3: Dog and Stranger (1st separation episode)	3-DS	The stranger continued to play with the dog if it was willing; if it was inactive or distressing, the stranger attempted to distract it with play or by providing verbal and tactile comfort
Episode 4: Dog and Owner (1st reunion episode)	4-DO	The owner entered the room and greeted and/or comforted his/her dog as usual after returning from work or shopping, while the stranger quietly left the room. The owner was free to play with the dog throughout the episode. At the end of this episode, the owner left the room
Episode 5: Dog alone	5-D	The dog remained alone for three minutes
Episode 6: Dog and Stranger (2nd separation episode)	6-DS	The stranger entered the room and followed the same procedure as in episode 3
Episode 7: Dog and Owner (2nd reunion episode)	7-DO	The owner entered the room greeted and followed the same protocol as in episode 4, while the stranger left the room

### Data collection and analysis

An ethogram containing 16 mutually exclusive behaviors was compiled (Table 2) and used for the collection of data throughout the SST. Dog behaviors were coded from the video by a trained observer using a 5-s instantaneous sampling method for the state events, using Solomon Coder beta® (ELTE TTK, Hungary). Stress behaviors of short duration (i.e., mouth licking, shaking, scratching itself, yawning, barking, yapping, ears back) were considered as discrete events and were recorded with a continuous sampling method. Nevertheless, these behaviors were only occasionally observed in some dogs and were not considered in the following analysis. Drinking during the SST was considered a stress-related behavior since dogs were allowed to drink ad libitum before the test.

**Table 2:** Behaviors recorded in the SST procedure

Behavior	Description
Drinking	Drinking from the water bowl
Exploration	Activity directed toward physical aspects of the environment including sniffing, visual inspection (e.g., implied the state of attention of the dog) and gentle oral examination such as licking
Locomotion	Walking, pacing, or running around, without exploring the environment or playing
Passiveness	Sitting, standing, or lying down without any obvious orientation toward the environment or person
Individual play	Any vigorous behavior or galloping movement directed toward a toy when not interacting with a person, including chewing, biting, shaking from side to side, scratching or batting with the paw, chasing rolling balls, and tossing using the mouth
Social play	Any vigorous behavior or galloping movement performed when interacting with either owner or stranger, including running, jumping, and chasing toys
Proximity seeking	Active proximity seeking behaviors, including approaching and following while clearly visually oriented towards the owner or stranger
Social interaction	Interaction with the person using actively a part of the body (e.g., by touching and pushing with the paw, muzzle or other parts of the body) excluding proximity seeking and social contact
Social contact	Being in physical contact with the person, excluding during greeting, social play and social interaction
Greeting	All greeting behaviors toward the entering owner or stranger, such as approaching, tail wagging, jumping, and physical contact. Greetings were allowed in the first 10 s (max 2 sampling points). Then the person was advised to invite the dog to play
Social avoidance	Actively avoiding an approaching person
Social gazing	Staring fixedly at the owner or stranger without any type of interaction
Interest in chair	The dogs gaze, sniff or enter in physical contact with the empty chair occupied previously by the owner or stranger
Approaching door	Actively approaching, while visually oriented to, the door
Gazing at the door	Visual orientation towards the door, when not approaching it
Physical contact door	All active behaviors resulting in physical contact with the door, including scratching the door with the paws, jumping on the door, and pulling on the door handle with the forelegs or mouth

All behaviors listed are mutually exclusive

The dataset contained 252 observations for each dog (12 points sample/min × 3 min × 7 episodes). For the inter-observer reliability, a second expert coder randomly analyzed a sample of 20 videos (about 40% of the sample). Both coders ignored the sex of the dogs during the coding. The behaviors were compared as a percentage of agreement between observers resulting in an agreement from 90 to 100% depending on the behavior observed. Thus, the data of the first coder were accepted and used for the statistical analysis.

Behaviors expressed by the dogs during the SST were analyzed through Principal Component Analysis (PCA), which aimed at both reducing the number of variables on which to look for sex differences and provide a better possibility to interpret such differences when found. Data were analyzed separately for Episode 1-DO and Episode 5-D since several behavioral variables (e.g., greetings and social play in both episodes, as well as any other social behavior in Episode 5-D) could not be expressed during these episodes. Attributing a frequency of 0 would have artificially inflated their weight in the PCA, and excluding them altogether would have made no sense, as they were crucial parts of the ethogram used in the SST. Since all variables could theoretically be expressed in all other episodes (2-DOS, 3-DS, 4-DO, 6-DS, 7-DO), these were analyzed together by a single PCA. Therefore, three separate PCA analyses were performed, one on behaviors expressed in 1-DO, one for 5-D, and one for all other episodes. Regarding the latter analysis, it must be noted that Episode 2-DOS differs from other episodes, for it entails the presence of both the owner and stranger and does not allow for distinguishing behaviors expressed towards one or the other. However, an analysis performed separately on Episode 2-DOS and Episodes 3, 4, 6 and 7 resulted in a substantially unchanged pattern of results, compared with the single analysis performed on all five episodes. Therefore, for the sake of simplicity and synthesis, only the latter is presented in the results section.

In all cases, all the variables regarding behaviors that could be expressed in the episodes were included in the initial PCA. Assessment of the Kaplan–Meyer–Olkin test for sampling adequacy was performed to determine the acceptability of the initial solution; if unsatisfactory, a stepwise exclusion of variables was performed until reaching a satisfactory value (i.e. *KMO* > *0.5*). The identification of factors was based on an *Eigenvalue* > *1*, and factor scores were calculated with the regression method.

The scores of factors obtained through the three PCAs were analyzed with a Generalized Linear Mixed Model (GLMM). Separate models were run for scores of each of the factors identified by each of the PCA, which were included in the model as a linear-dependent variable. The model included the dog’s sex and age and the owner’s sex as fixed factors; moreover, for the analysis of factors obtained from the PCA on multiple episodes, the Episode was also included as a fixed factor. In the latter case, first-order interactions between the Episode and the other fixed factors (dog’s sex, dog’s age, owner’s sex), were also included in the model. Finally, the model included the dog’s name as a random factor, accounting for the covariance of measures taken from the same dog across the episodes of the SST. Post hoc contrasts were computed with Bonferroni correction whenever a significant effect was found for a factor included in the model.

## Results

The mean frequencies of the behaviors expressed by dogs in each SST episode are reported in Table 1 of Online Resource 1. The final PCA on the behaviors expressed in 1-DO obtained a *KMO* = *0.501*. It resulted in the identification of three factors, which overall explained 68.7% of the variance. The loadings of each behavioral variable are reported in Table 2 of Online Resource 1. Factor 1 identified a proximity-seeking dimension; Factor 2 included engagement in non-social activities (alternatively exploration or social play, which loaded with opposite signs on the factor); Factor 3 described dogs’ motivation to leave the room. The GLMM model did not find any effect of sex, age, or the owner’s sex on any of the three factors (Table 3).

**Table 3:** The results of the Generalized Linear Mixed Model, indicating the effect of the episode, the dogs’ sex and age, and of the owner’s sex on the factors’ scores for Episode 1-DO, for Episode 5-D and Episodes 2–7 of the SST

Episode(s)	Model term	Degrees of freedom	Factor 1		Factor 2		Factor 3		Factor 4	
			*F*	*P*	*F*	*P*	*F*	*P*	*F*	*P*
Episode 1	Dog sex	1	1.09	0.30	0.73	0.83	0.88	0.35	–	–
	Dog age	1	0.12	0.73	0.26	0.40	0.35	0.55	–	–
	Owner sex	1	0.31	0.58	0.04	0.62	0.05	0.82	–	–
Episode 5	Dog sex	1	0.36	0.54	0.01	0.91	–	–	–	–
	Dog age	1	0.45	0.50	1.72	0.09	–	–	–	–
	Owner sex	1	0.27	0.60	3.11	0.19	–	–	–	–
Episodes 2–7	Episode	1	**43.28**	** < 0.01**	**8.62**	** < 0.01**	**5.28**	** < 0.01**	1.47	0.22
	Dog sex	1	0.53	0.46	**11.98**	** < 0.01**	2.81	0.09	2.28	0.13
	Dog age	1	0.81	0.37	0.39	0.53	1.08	0.29	**12.94**	** < 0.01**
	Owner sex	1	0.15	0.69	3.14	0.08	0.03	0.85	0.10	0.74
	Episode*Dog sex	4	**2.71**	**0.03**	1.96	0.10	**2.58**	**0.04**	0.48	0.74
	Episode*Dog age	4	**3.92**	** < 0.01**	1.32	0.26	0.42	0.79	**4.44**	** < 0.01**
	Episode*Owner sex	4	0.64	0.63	0.91	0.45	0.44	0.77	1.41	0.23

Bold types indicate significant effects

The final PCA on the behaviors expressed in 5-D obtained a *KMO* = *0.620*. It resulted in the identification of two factors, which overall explained 59.8% of the variance. The loadings of each behavioral variable are reported in Table 3 of Online Resource 1. Factor 1 appears to represent a dimension of distress, while Factor 2 depicted engagement in individual activities. The GLMM model did not find any effect of sex, age, or the owner’s sex on any of the two factors (Table 3).

The final PCA on the behaviors expressed in all other episodes (2-DOS, 3-DS, 4-DO, 6-DS, 7-DO, hereafter collectively referred to as Episodes 2–7) obtained a *KMO* = *0.52*. It resulted in the identification of four factors, which overall explained 51.0% of the variance. The loadings of each behavioral variable are reported in Table 4 of Online Resource 1. Factor 1 was the most complex, comprising four variables with high loadings, and possibly reflecting different interrelated behavioral dimensions. Its interpretation requires some considerations, which are presented in the Discussion section. Factor 2 was constituted by social behaviors with positive loadings, clearly representing the social proximity seeking dimension of dogs’ behavior in the SST. Factor 3 comprised behaviors directed to the chairs or towards the door, suggesting the factor represents dogs’ motivation to seek for the person. Factor 4 seems to reflect social disinterest. Although passivity was earlier considered by some authors as an indicator of a secure-base effect (Palmer and Custance 2008), others considered this behavior as an active suppression of behavioral signs rather than a relaxed reaction to social challenges (Gácsi et al. 2001; Mongillo et al. 2013). Its association with social avoidance in our PCA supports the latter view.

Results of the GLMM indicating the effect of the SST episode, the dogs’ sex and age, the owner’s sex, and their interaction with the SST episodes on the factors identified by the PCA are reported in Table 3.

**Fig. 1:**
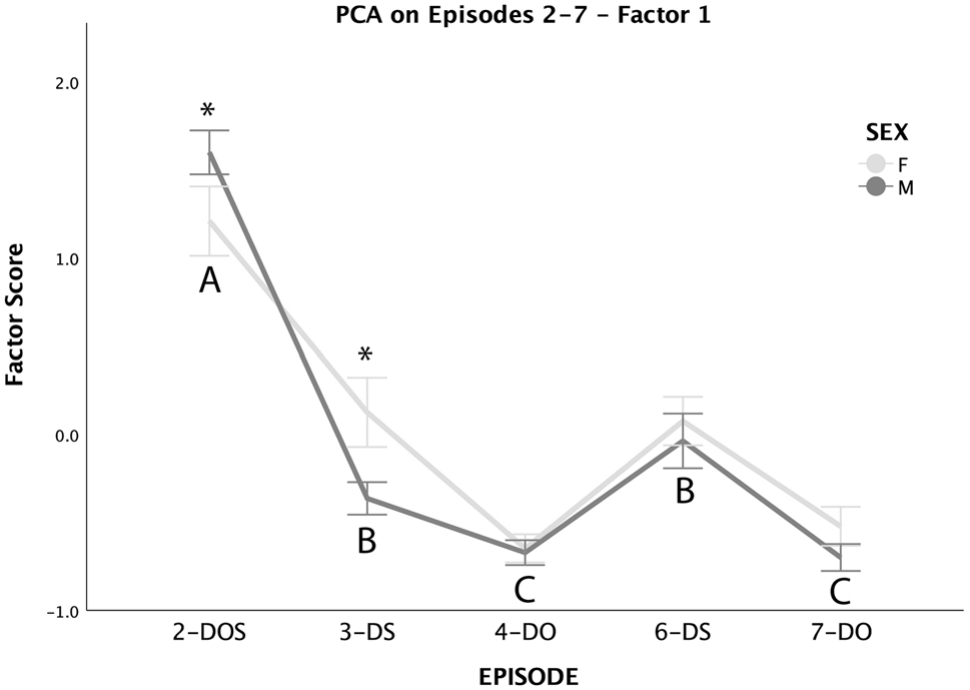
Mean ± SE scores of Factor 1 obtained by male (dark gray) and female (light gray) dogs across Episodes 2–7 (excluded Episode 5–dog alone). Different capital letters indicate significant differences in scores obtained in different episodes, regardless of the dogs’ sex. Significant differences between males’ and females’ scores within specific episodes are flagged by an asterisk (sequential Bonferroni-corrected post hoc comparison after Generalized Linear Mixed Model). *DOS* dog, owner and stranger, *DS* dog and stranger, *DO* dog and owner

Scores of Factor 1 were affected by the episode, by the interaction between episodes and the dog’s sex, and by an interaction between episode and age. The trend across the episodes was similar for males and females, with the highest scores at the beginning of the test (2-DOS) and lowest when dogs were reunited with their owner (4-DO and 7-DO); however, females obtained significantly lower scores than males in 2-DOS, but higher scores than males in 3-DS (Fig. 1). In regards to the interaction of episode and age, scores slightly decreased with increasing age for Episode 2-DOS (*r*^*2*^ = 0.06), but had an opposite, increasing trend for episodes 3-DS and 6-DS (*r*^*2*^ = 0.09 for both), and were substantially unaffected by age in episodes 4-DO and 7-DO (*r*^*2*^ < 0.02 for both).

**Fig. 2:**
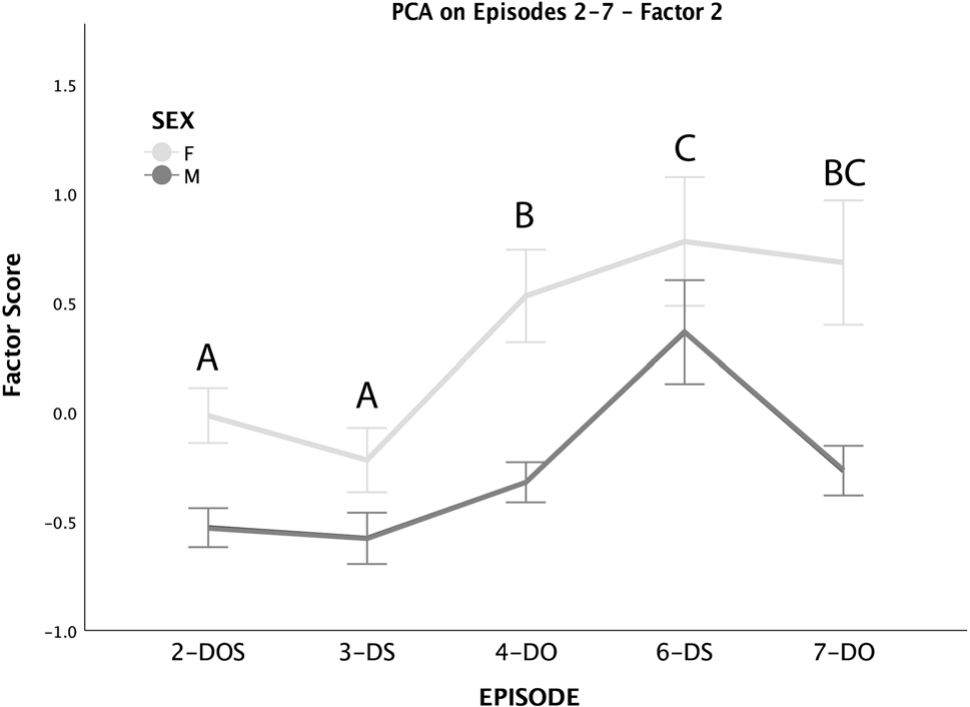
Mean ± SE scores of Factor 2 obtained by males (dark gray) and female (light gray) dogs across Episodes 2–7 (excluded Episode 5–dog alone). Different capital letters indicate significant differences in scores obtained in different episodes, regardless of the dogs’ sex. Overall, male scores were significantly lower than those of females, regardless of the episode. The score of episodes in which two letters are reported is not significantly different from that of other episodes in which any of the two letters appear (sequential Bonferroni-corrected post hoc comparison after Generalized Linear Mixed Model). *DOS* dog, owner and stranger, *DS* dog and stranger, *DO* dog and owner

Scores for Factor 2 were affected by episodes, with lower scores found in 2-DOS and 3-DS (Fig. 2). There was also a main effect of dog’s sex, whereby females obtained higher scores than males; the difference appeared particularly evident in episodes in which the dog was with the owner (estimated mean with 95% confidence intervals Episode 4-DO: Females = 0.529 (0.148–0.910) vs. Males = − 0.314 (− 0.645–0.017); estimated mean with 95% confidence intervals Episode 7-DO: Females = 0.682 (0.301–1.063) vs. Males = − 0.261 (− 0.592–0.070)), rather than with the stranger (3-DS: Females = − 0.221 (− 0.601–0.160) vs. Males = − 0.571 (− 0.902 to − 0.240); 6-DS: Females = 0.779 (0.398–1.159) vs. Males = − 0.372 (0.041–0.703)), although the interaction between episode and dog’s sex did not result in a statistically significant difference.

**Fig. 3:**
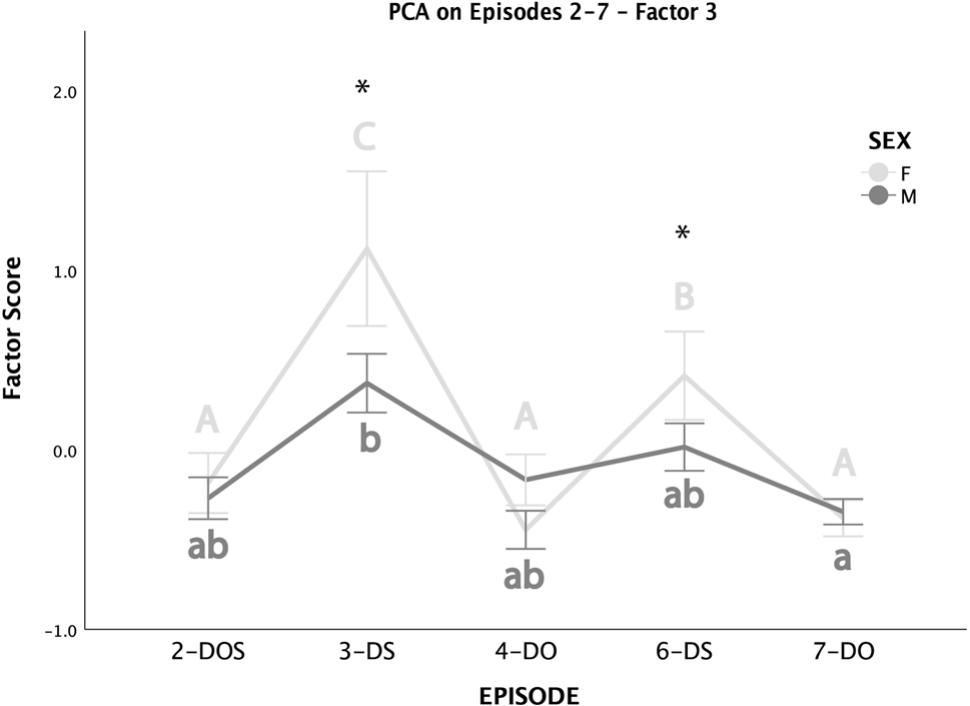
Mean ± SE scores of Factor 3 obtained by male (dark gray) and female (light gray) dogs across Episodes 2–7 (excluded Episode 5–dog alone). Different capital letters indicate significant differences in scores obtained in different episodes by females. Different small-cap letters indicate significant differences in scores obtained in different episodes by males. In both cases, the score of episodes in which two letters are reported is not significantly different from that of other episodes in which any of the two letters appear. Significant differences between males’ and females’ scores within specific episodes are flagged by an asterisk (sequential Bonferroni-corrected post hoc comparison after Generalized Linear Mixed Model). *DOS* dog, owner and stranger, *DS* dog and stranger, *DO* dog and owner

Scores of Factor 3 were affected by both the episode and by the interaction between episodes and dog’s sex. Females obtained the highest score in 3-DS, followed by 6-DS (the two dog-stranger episodes), in which scores were significantly higher than when the dog was with its owner (4-DO and 7-DO) or with both (2-DOS). Scores obtained by males were less variable, with the only significant difference being found for Episode 3-DS which obtained a higher score than Episode 7-DO. The scores of 3-DS and 6-DS were also significantly higher in females than in males (Fig. 3).

Scores for Factor 4 were affected by the dog’s age and by the interaction between episodes and the dog’s age. Specifically, scores increased as a function of age in Episode 2-DOS (*r*^*2*^ = 0.24), while the increase in all other episodes was much less steep (*r*^*2*^ between 0.03 and 0.08).

## Discussion

This study aimed at detecting differences between male and female dogs in the expression of attachment-related behaviors, in a widely used SST procedure. The analytical approach entailed a PCA on the frequency of behaviors expressed by dogs in different SST episodes and subsequent exploration of sex differences on the factors identified by such PCA; also, the dog’s age and the owner’s sex were explored as factors of interest.

No differences between sexes were detected in the factors obtained by the PCA for Episode 1-DO or Episode 5-D. Although in different respects, the two episodes are less informative than others on social-related behaviors. Indeed, behaviors expressed in the first episode are largely conditioned by the arrival in a new unfamiliar place, resulting in exploration being the predominant behavior. Our results indicate that no difference exists between males and females in this respect. Episode 5-D, on the other hand, is characterized by the absence of any social stimulus; therefore the socially driven difference between males and females would not be evident here. Nonetheless, the absence of differences between sexes suggests that male and female dogs cope similarly with being left alone in an unfamiliar place.

Before discussing differences related to sex and the other variables of interest on Factors resulting from the PCA on Episodes 2–7, a first consideration needs to be done on Factor 1 of such analysis. This factor’s score possibly reflects different interrelated behavioral dimensions. On one hand, the factor may represent dogs’ proneness to engage in non-social activities, such as play and exploration, stimulated by the novel environment and the presence of toys. On the other hand, the factor is likely linked to distress, as it includes aimless locomotion. It is noteworthy that the high positive loading of individual play also fits within a stress-related dimension, as it was shown that dogs express individual play as a coping mechanism in the SST (Scandurra et al. 2016). The same interpretation may hold also for exploration: traditionally exploration expressed in the presence of the attachment figure has been associated with a secure-base effect in humans (Ainsworth 1989), chimpanzees (Ainsworth and Bell 1970; Ainsworth et al. 1978; Bard 1991), and dogs (Gácsi et al. 2001; Palmer and Custance 2008). However, a recent paper found no difference in exploration levels between stranger and owner, when the order of episodes was inverted between the two figures (Rehn et al. 2013). Therefore, exploratory behavior does not supply convincing evidence of a secure-base effect. The current PCA included behaviors recorded from the second episode onwards, when, theoretically, dogs had already acquired most environmental information. In this sense, exploration could more likely be related to stress due to exposure to a novel, unfamiliar environment. For example, in an unfamiliar environment, exploration behavior positively correlates with neophobia, and factors increasing anxiety also increase exploration (Russell 1973). If this interpretation is correct, then Factor 1 represents a behavioral dimension linked to stress. The interpretation is further supported by the negative loading of social play, a behavior for which negative correlations with stress-related behaviors were reported for both children (Ainsworth et al. 1978) and dogs (Horváth et al. 2008; Prato-Previde et al. 2003; Schöberl et al. 2016).

The score of Factor 1 was overall higher in Episode 2-DOS than all other episodes. This is not surprising since in this episode people were asked to ignore the dogs for the first 2 min. The factor received lower average scores when the owner was present than when the stranger was present, again highlighting the stress-related dimension captured by the factor, and in agreement with the attachment theory forecasting increased signs of distress when separations from the caretaker occur (Bowlby 1958, 1969). Such pattern of scores across the episodes was similar in males and females, indicating that both males and females showed different behaviors when in the presence of the owner than when with the stranger. However, males obtained a higher score in Episode 2-DOS but lower scores in Episode 3-DS, when the owner left the room for the first time. This result may indicate that males cope better with the stressful context in the first episode of separation. Interestingly, while systematic investigations on sex differences in coping with stress in animals are scarce, the idea that in adulthood males may show better coping with distress is also suggested by studies in both rodents and humans (Bale and Epperson 2015).

Scores of Factor 2 were generally lower at the beginning of the test—where the possibility for interaction between the dog and people was limited by the procedure—and increased across the episodes, without a clear dependency on the presence of the owner. Therefore, the score does not seem to reflect any specific owner- or attachment-related dimensions but more likely reflects a general motivation for social interaction. Such social interest was overall higher in females, with no significant differences between males and females in how the score evolved through the SST, nor with the difference in any specific episode. Therefore, the result indicates that females show overall higher sociability than males, which agrees well with both popular beliefs as well as previous scientific reports. Indeed, females appeared generally more sociable in other studies (Lore and Eisenberg 1986; Wilsson and Sundgren 1997), including a higher soliciting of cooperative behaviors by strangers, than that expressed by males (Persson et al. 2015).

Scores of Factor 3 were higher in episodes when the dogs were with the stranger (Episodes 3-DS and 6-DS), clearly making this a factor reflecting dogs’ motivation to reunite with the owner, one of the key features of the attachment. Of relevance to our aims, the factor showed a much higher variation in females than in males; indeed, only in females the difference between the score obtained in separation episodes was consistently higher than when the owner was present. Moreover, the score was higher in females than males in the first episode of separation. Therefore, the results indicate females are more susceptible than males to separation from the attachment figure when left with the stranger, in turn suggesting that the male and female dogs differ in how they express attachment-related behaviors.

Most of the studies that, among other factors, explored the effect of sex on dogs’ behavior in the SST, report no differences between males and females. With many potentially intervening variables, it is difficult to determine why we did find sex-related differences while several other studies did not. Possible reasons include relevant differences in the sampled population. For instance, some of the mentioned studies tested sheltered dogs (Gácsi et al. 2001) or guide dogs (Fallani et al. 2006), which clearly cannot be compared with pet dogs. In other cases, there are substantial differences in the statistical approach. For instance, in the remarkable work by Topál and colleagues (1998), relatively few behavioral variables were collected and the analysis did not take into account their expression across different episodes. Moreover, even when collected variables were more similar to those of the current study, the sample size may not have been sufficient to detect sex differences (Prato-Previde et al. 2006); indeed, when a similar data collection was applied to a slightly larger sample, some, albeit small, differences between sexes emerged (Prato-Previde et al. 2003). Finally, unlike most other studies, our sample involved dogs of a single breed, Labrador Retrievers. While this also represents a potential limitation of this study (as discussed below), this choice removed breed-related variability and increased the possibility of our procedure to highlight sex-related differences.

Relevant to our findings is the general agreement that no sex-related differences exist in the behavior of human infants in the SST (Del Giudice 2019). Sex differences in attachment behavior have been reported in humans, but it is not until mid-childhood that they start to emerge, eventually becoming full-fledged in adult romantic relationships (Del Giudice 2019). Within these relationships, sex differences are generally characterized by greater self-reliance and avoidance of the attachment figure by men, and greater anxiety (preoccupation and neediness) by women (Bartholomew and Horowitz 1991; Del Giudice 2019). Although a full comparison of dogs’ behavior in the SST and the dog–human relationship with adult human attachment would be inappropriate at this stage, there is some suggestive similarity between the two situations. Specifically, female dogs showed more owner reunion-seeking behaviors following the separation from the attachment figure than males did. At the same time, males showed a lower motivation toward social contacts, which, although not specific to the owner, could reflect a social avoidance component. These sexually dimorphic manifestations seem therefore to parallel the avoidance and the anxiety dimensions that predominate in romantic relationships in men and women, respectively. Del Giudice and Belsky (2010) placed the origin of human sex differences in attachment into an evolutionary framework. According to their view, the emergence of such differences is paired to the juvenile onset of competition among peers for social status and intensification of adult, sexually differentiated behaviors during social play, including for instance increased aggression by boys and parenting behavior by girls. In this sense, avoidance in the attachment relationship along with aggression and inflated self-esteem, are considered parts of a status‐seeking strategy for young males, tuned to increased mating efforts, early reproduction, and selfish risk‐taking. Interestingly, both aggression and boldness are also generally more prominent in male dogs than bitches (Scandurra et al. 2018b). In regard to girls, anxiety may also have a functional role in the maintenance of social relationships, through closeness-seeking and dependent behaviors (Del Giudice and Belsky 2010). Again, a similarity may be found here with dogs, where females are generally more prone to social contact and interaction than males are (Scandurra et al. 2018b). In summary, although we cannot fully demonstrate that the same framework in which sex differences in human adult attachment relationships are explained applies to dogs, there is some suggestive evidence that points in this direction.

Besides sex, age also affected the score of some of the Factors of the PCA for Episodes 2–7. Specifically, scores of Factor 1 increased with increasing age in episodes when the dog was left with the stranger. This effect could be related to the development of a more selective relationship with the owner with increasing age (Valsecchi et al. 2010). However, the lack of age’s effects in episodes in which the dog was alone with the owner supports an alternative view that older dogs cope less efficiently with the emotional distress caused by the SST while keeping attachment-related behaviors unaltered (Mongillo et al. 2013). The effect of age was also evident in the social disinterest dimension, particularly in the second episode of the SST. As for most of this episode, dogs could not interact with either the owner or stranger. Factor 4 seems to reflect older dogs’ lower motivation to perform individual activities, which in this circumstance included play and exploration. As opposed to the results reported by Mongillo and collaborators (2013), we did not find a clear indication that passive behavior increased more during separation episodes for older than for younger dogs, compared to the initial episodes of the SST. It should be noted, however, that our study was not designed to tackle age differences, and our sample included only a relatively small number of older dogs.

This study presents some limitations. First, in our experimental procedure a man played the part of the stranger; the reason for involving a single person was to increase the standardization of his behavior and limit variability. While in most previous studies that looked at dogs’ behavior in the SST a woman acted as the stranger, no clear indications exist in the literature in favor or against the involvement of strangers of either gender or its potential effects on dogs’ responses. Parthasarathy and Crowell-Davis (2006) used strangers of both sexes and found no effects of gender on the behavior of dogs. However, some variables used in such study were slightly different from ours, not allowing us to apply the concept tout-court to the present study. Notably, the human literature presents a similar dilemma. Most studies employed woman as strangers. Only a few papers systematically investigated the effect of the stranger’s gender on children’s reactions in the SST and provided inconsistent results (Batter and Davidson 1979). In view of these considerations, it would be important as a future perspective to investigate if and how the stranger’s gender, possibly also in relation (same/opposite) with the owner’s gender, could have any effect on the behavior of male and female dogs. A second limitation of our study is that our sample included only a limited number of gonadectomized animals, with an imbalanced distribution between the two sex groups. This prevented us to assess the role of sex hormones in the expression of behaviors. Because of the relevance in social and affective behavior of hormonal effects observed in other species, including humans, it will be important in future studies to address this specific aspect. Finally, as stated before, our results were obtained in a single-breed sample of Labrador Retriever dogs, mainly with the aim of limiting breed-specific effects. In this sense, however, results cannot be generalized, given all reported behavioral breed differences (Mehrkam and Wynne 2014) and, specifically, in the SST where, for instance, Golden Retrievers appeared shyer and more insecure than Labrador Retrievers (Fallani et al. 2006). Thus, studies extended to other breeds are required for a robust generalization of sex differences in the SST.

## Conclusion

This study reports the first evidence of differences between female and male dogs in the expression of dog-owner attachment behaviors in the SST. The lack of corresponding differences in human’s infant-mother attachment behavior, but similarities with sex differences reported in human romantic attachment suggests that the dog-owner bond could be characterized by aspects that are typical of adult human relationships. The latter concept was recently also proposed by Savalli and Mariti (2020), who identify the dog-owner bond as resembling a friendship between adult humans. This does not imply that the infant-mother attachment theory is no longer a valid model for the dog-owner bond. However, it suggests that such a bond may be not fully captured by parallels with the human infant-mother relationship and prompts to inquire into those aspects.

## Supplementary Information

Below is the link to the electronic supplementary material.

Supplementary file1 (DOCX 45 KB)

## Data Availability

All tables and graphical data obtained during this study are included in this published article and its Supplementary Information file (see Supplementary Data).
