# Supplementary material for: Is this love? Sex differences in dog-owner attachment behavior suggest similarities with adult human bonds
Source: Anim Cogn. 2021 Aug 5;25(1):137–48. doi: 10.1007/s10071-021-01545-w (PMC8904329; doi:10.1007/s10071-021-01545-w)
Supplement: Supplementary file 1 — Supplementary file1 (DOCX 45 KB) [file 10071_2021_1545_MOESM1_ESM.docx]

**ANIMAL COGNITION**

**Is this love? Sex differences in dog-owner attachment behavior suggest similarities with adult human bonds**

Biagio **D’Aniello^a,*^,** Anna **Scandurra^a^,** Claudia **Pinelli^b^,** Lieta **Marinelli^c^** & Paolo **Mongillo^c^**

^a^Department of Biology, University of Naples Federico II, via Cinthia, 80126, Naples, Italy.

^b^Department of Environmental, Biological and Pharmaceutical Sciences & Technologies, University of Campania “Luigi Vanvitelli”, Caserta, Italy.

^c^Laboratory of Applied Ethology, Department of Comparative Biomedicine and Food Science, University of Padua, Viale dell’Università 16, Legnaro, PD 35020, Italy.

***Corresponding author**: Biagio D’Aniello

**e-mail**: biagio.daniello@unina.it

**Table 1** Mean±SD number of sample points for which each behavior was observed across the SST episodes; empty cells represent behaviors that could not be expressed in a certain episode.

| **Behavior** | **Episode** | | | | | | |
| --- | --- | --- | --- | --- | --- | --- | --- |
|  | 1 | 2 | 3 | 4 | 5 | 6 | 7 |
| Approaching door | 0.3±0.6 | 0.3±0.6 | 0.4±0.7 | 0.2±0.5 | 0.7±0.9 | 0.4±0.7 | 0.1±0.3 |
| Drinking | 1.8±2.2 | 0.6±1.2 | 0.3±1.0 | 0.5±1.0 | 0.6±1.3 | 0.4±1.0 | 0.4±1.0 |
| Exploration | 10.9±8.3 | 4.1±4.1 | 0.6±1.3 | 0.0±0.2 | 2.8±3.0 | 0.8±1.7 | 0.4±1.0 |
| Gazing at the door | 2.7±4.4 | 1.1±2.1 | 5.6±8.1 | 0.4±0.7 | 24.0±8.0 | 6.9±9.0 | 0.4±1.3 |
| Greeting |  | 0.4±0.7 | 0.0±0.0 | 1.1±1.0 |  | 0.8±0.8 | 1.1±0.8 |
| Individual play | 6.3±8.7 | 4.7±5.9 | 0.8±2.6 | 0.3±1.1 | 0.6±1.7 | 0.7±2.1 | 0.2±0.8 |
| Interest in chair | 0.0±0.1 | 0.0±0.1 | 0.4±1.3 | 0.0±0.1 | 2.2±3.5 | 0.1±0.5 | 0.0±0.0 |
| Locotion | 3.6±3.5 | 2.2±2.5 | 0.6±1.2 | 0.2±0.6 | 1.3±1.7 | 0.7±1.6 | 0.2±0.6 |
| Passiveness | 4.8±7.4 | 4.7±7.1 | 0.4±1.3 | 0.3±1.1 | 2.1±6.0 | 1.4±3.7 | 0.4±2.0 |
| Physical contact door | 0.0±0.3 | 0.0±0.1 | 0.1±0.5 | 0.0±0.0 | 0.6±1.9 | 0.1±0.4 | 0.0±0.0 |
| Proximity seeking | 0.8±1.1 | 1.5±1.2 | 0.6±1.2 | 0.4±0.8 |  | 1.5±2.7 | 0.4±1.0 |
| Social avoidance | 0.0±0.0 | 0.0±0.3 | 0.2±1.5 | 0.0±0.0 |  | 0.2±0.7 | 0.0±0.0 |
| Social contact | 0.0±0.0 | 0.6±1.4 | 1.6±2.7 | 1.5±2.8 |  | 3.2±5.2 | 2.3±4.7 |
| Social gazing | 2.2±4.1 | 3.4±4.3 | 0.6±1.2 | 0.4±0.8 |  | 1.5±2.7 | 0.4±1.0 |
| Social interaction | 1.4±3.5 | 1.9±3.9 | 0.0±0.0 | 0.0±0.0 |  | 0.0±0.0 | 0.0±0.0 |
| Social play | 0.0±0.0 | 9.5±3.8 | 20.9±13.7 | 30±6.1 |  | 13.9±13.8 | 29.2±8.2 |

**Table 2** Loadings in the factors identified by PCA for the frequencies of behaviors expressed during Episode 1-DO (loadings < 0.4 are not listed in the table, as are behaviors that did not obtain a loading > 0.4 in any of the 3 factors).

| **Behavior** | **Factor** | | |
| --- | --- | --- | --- |
|  | 1 | 2 | 3 |
| Social interaction | 0.965 |  |  |
| Proximity seeking | 0.957 |  |  |
| Exploration |  | -0.789 |  |
| Individual play |  | 0.774 |  |
| Approach door |  |  | 0.794 |
| Physical contact door |  |  | 0.713 |

**Table 3** Loadings in the factors identified by PCA for the frequencies of behaviors expressed during Episode 5-D (loadings < 0.4 are not listed in the table, as are behaviors that did not obtain a loading > 0.4 in any of the 2 factors).

| **Behavior** | **Factor** | |
| --- | --- | --- |
|  | 1 | 2 |
| Locomotion | 0.797 |  |
| Approaching door | 0.746 |  |
| Drinking | 0.647 |  |
| Passiveness | 0.581 |  |
| Exploration |  | 0.819 |
| Individual Play |  | 0.812 |

**Table 4** Loadings in the factors identified by PCA for the frequencies of behaviors expressed during Episodes 2-7 (loadings < 0.4 are not listed in the table, as are behaviors that did not obtain a loading > 0.4 in any of the four factors).

| **Behavior** | **Factor** | | | |
| --- | --- | --- | --- | --- |
|  | 1 | 2 | 3 | 4 |
| Social play | -0.736 |  |  |  |
| Locomotion | 0.684 |  |  |  |
| Exploration | 0.626 |  |  |  |
| Individual play | 0.590 |  |  |  |
| Social contact |  | 0.818 |  |  |
| Greeting |  | 0.619 |  |  |
| Proximity seeking |  | 0.523 |  |  |
| Approaching door |  |  | 0.696 |  |
| Interest in chair |  |  | 0.688 |  |
| Passiveness |  |  |  | 0.650 |
| Social avoidance |  |  |  | 0.648 |
